# Development, Characterization and Optimization of the Anti-Inflammatory Influence of Meloxicam Loaded into a Eucalyptus Oil-Based Nanoemulgel

**DOI:** 10.3390/gels8050262

**Published:** 2022-04-22

**Authors:** Tamer M. Shehata, Hanan M. Elnahas, Heba S. Elsewedy

**Affiliations:** 1 Department of Pharmaceutical Sciences, College of Clinical Pharmacy, King Faisal University, Alhofuf 31982, Saudi Arabia; helsewedy@kfu.edu.sa; 2 Department of Pharmaceutics and Industrial Pharmacy, Faculty of Pharmacy, Zagazig University, Zagazig 44519, Egypt; hananelnahas@gmail.com

**Keywords:** meloxicam, nanoemulgel, anti-inflammatory, eucalyptus oil, optimization

## Abstract

The purpose of the present study was to explore the influence of a certain natural essential oil, namely eucalyptus oil, as an anti-inflammatory agent in addition to its prospective role in enhancing the action of meloxicam in reducing inflammation. As far as we know, this has been the first integration of meloxicam and eucalyptus essential oil into a nanoemulgel formulation intended for topical use. Primarily, eucalyptus oil was utilized in developing a nanoemulsion formulation incorporating meloxicam. A 2^2^ factorial design was constructed using two independent variables (oil concentration and surfactant concentration) with two responses (particle size and % of in vitro release). One optimized formula was selected depending on the desirability function and subjected to a stability study. The optimized nanoemulsion was mixed with HPMC as a gelling agent to produce a meloxicam-loaded nanoemulgel, which was examined for its properties, stability, in vitro release and ex vivo permeation. Eventually, the anti-inflammatory activity was evaluated and compared with a placebo and corresponding gel formulation. The developed nanoemulgel revealed acceptable physical characteristics to be applied topically. Studying of the in vitro release was conducted successfully for 6 h. The ex vivo permeation from the nanoemulgel formulations was prompted, showing an appropriate value of the steady-state transdermal flux (SSTF). As a final point, the anti-inflammatory activity of the developed nanoemulgel revealed a valued anti-inflammatory influence. Additionally, the concurrence of eucalyptus essential oil and meloxicam was assured, and their potential in combating and lowering inflammation was supported.

## 1. Introduction

Conventional dosage forms intended for transdermal and topical application were greatly applied formerly, including ointments, creams and patches; however, certain weaknesses have limited their administration. Among these problems are poor solubility, bad penetration and an inadequate drug-loading capacity, in addition to certain stability problems and poor spreadability, which necessitate overcoming these drawbacks to reach more comfortable and reproducible activity [1].

Nanotechnology is an advanced approach, intended for design, and it develops matters in a nanoscale range, acting to address the undesired properties of active constituents and maximize their therapeutic effects [2]. Several nanocarriers can be applied for these purposes, namely liposome, ethosome, niosome, nanoparticles and a nanoemulsion [3].

A nanoemulsion (NE) is one of the lately developed nanocarriers that have gained a lot of attention owing to various merits, mostly their small particle size, which would provide better absorption and, consequently, improve the bioavailability of the incorporated drug [4]. Moreover, it could improve drug solubility, offer controlled release of drug and provide protection against degradation [5]. NE is a thermodynamically stable system formed of an aqueous phase, oily phase, surfactant and sometimes co-surfactant to form a single-phase system [6]. It exhibits more stability over conventional emulsions in terms of flocculation, phase separation, sedimentation and creaming [7]. It is a promising drug delivery system, which could be administered via different routes of administration, including parenteral [8], oral [9] and transdermal routes [10]. However, for a transdermal delivery of the drug, it is practically better to incorporate the developed nanoemulsion into a hydrogel base to form a novel dosage form, termed a nanoemulgel (NEG).

NEG is a contemporary formulation anticipated for treating skin disorders. It can enhance permeability of the drug through the skin [11] in addition to its good rheological properties and the dual effects of both the nanoemulsion and hydrogel [12]. It can provide better drug adhesion to the skin, which leads to a higher concentration gradient towards the skin. Additionally, integrating a drug into an NEG formulation could improve its stability and ensure a controlled release [13]. Nowadays, NEGs have been involved in the treatment of several infections incorporating a variety of agents, such as antibacterial, antifungal, anticancer and anti-inflammatory agents [14].

Presently, the treatment of several diseases has been focused on exploring natural products, owing to their safety and evidenced pharmacological activities [15]. Eucalyptus is a natural medicinal plant that belongs to the Myrtaceae family, renowned for its essential oils that have been extracted from *Eucalyptus globulus* and have shown great potential in the field of nanotechnology [16]. Eucalyptus essential oil (EEO) has proven to be effective in treating certain skin disorders, such as dermatitis [17]. In addition, it has exhibited antioxidant, antibacterial and antifungal behavior [18]. Its main constituent is known as Eucalyptol (1,8-cineole), which has been proven to show analgesic and anti-inflammatory influences, since it inhibits the synthesis of certain cytokines in inflammatory cells [19,20]. EEO incorporated into various nanocarriers, such as a microemulsion, nanoemulsion and nanoemulgel, has confirmed its improved performance and, consequently, augmentation of the therapeutic influence [21,22].

Meloxicam (MX) is regarded as one of the potent non-steroidal anti-inflammatory drugs (NSAIDs) related to the enolic acid group and proposed for treating various inflammatory disorders [23]. It exerts a potent inhibition of cyclo-oxygenaze-2 more than cyclo-oxygenaze-1, which results in an identical effectiveness as other NSAIDs, however, with lower toxicity [24]. Owing to the recurrent gastrointestinal complications that have been associated with the oral administration of MX, other alternative routes of administration seem to be necessary. Therefore, delivering MX through the skin via incorporation into a transdermal formulation has been viewed as one of the supportive ways to evade the disadvantages of oral administration, gain the benefits of applying the drug directly to the target site and lessen the dosing frequency, which would improve the compliance of the patient [25,26]. There have not been several studies that have investigated the influence of MX when incorporated into a nanoemulgel formulation; however, Drais et al. used almond and peppermint oil for preparing an MX-NEG [27]. As far as we know, no previous literature has investigated the synergistic effect between MX and eucalyptus essential oil for boosting the anti-inflammatory effect.

For a more proficient study, a specific trend could be employed in order to design, optimize and examine the interrelation between certain factors and their related responses, which is well-known as quality by design (QbD) [28]. Central Composite Design (CCD) is one of these tools that help in such optimization, depending on specific mathematical equations and statistical analyses for inspecting the model [29].

In these contexts, MX loaded into various EEO-based NEs was developed through employing a 2^2^ full factorial design and investigating the influence of specific variables on the NE characterization. At that point, one optimized NE formulation was selected and integrated with the hydrogel base and went through definite physical and chemical evaluations. To conclude, the anti-inflammatory activity of the developed MX-NEG containing EEO was investigated to validate its potential as an efficient anti-inflammatory agent. 

## 2. Results and Discussion

### 2.1. Solubility Examination of MX in Formulation Constituents

The solubility of MA in various components was evaluated and found to be 216 ± 11, 130 ± 10, 68 ± 6 and 146 ± 5 mg/mL in EEO, tween 80, transcutol^®^ P and PEG 400, respectively. These results were matched with previous findings obtained in an earlier study [30].

### 2.2. Experimental Design

#### 2.2.1. Fitting the Model

CCD software (version: 12.0 software (Stat-Ease, Minneapolis, MN, USA)) is a tool that was used to create a design matrix with 11 investigates divided into four factorial points, four axial points and three central points. Table 1 summarizes the value and influence of each independent variable and observed response for different nanoemulsion formulations.

#### 2.2.2. Statistical Data Analysis

A number of homogenous EEO-based NE formulations incorporating MX were developed, and all appeared to be stable with no sign of phase separation at room temperature. The statistical analysis of the data was very essential for exploring the results and identifying the model. Predominantly, the quadratic model appeared to be the most fitted one if compared to other models, since this model maximized the values of the adjusted and predicted R^2^. As shown in Table 2, the obtained *p*-values of the two responses, Y_1_ and Y_2_, remained less than 0.0001, which emphasizes the significant effects of the independent variables on the observed responses [31]. It is generally known that the model terms are considered to be statistically significant if the *p*-value is less than 0.05. Referring to the obtained data, in the case of Y_1_, the terms A, B, AB, A^2^ and B^2^ were revealed to be significant, in addition to A, B and A^2^ related to Y_2_, which were proven to be significant. Furthermore, higher F-values are more preferable, as more error in the model can be attained from lower F-values of the responses. The current data indicated that the model F-values in all responses appeared to be significant. A lack of fit is other constraint that helps to checking if the data are fitted to the model, and as a consequence, a non-significant lack of fit is indispensable in order to fit the model [32]. The detected responses showed non-significant lack of fit values (*p* > 0.05) of 0.1255 and 2.00 with corresponding *p*-values of 0.9369 and 0.3501 for Y_1_ and Y_2_, respectively.

### 2.3. Characterization

#### 2.3.1. Effect of Independent Variables on Particle Size Determination

As the particle size of NE represents a crucial variable in its stability, it was very important to estimate [33]. As shown in Table 1, it was apparent that the developed NEs showed a particle size in the nano range (139 ± 2.31 to 257 ± 3.61). Referring to the data, it was noticeable that upon increasing the oil concentration, an increase in the particle size of the NE formulation was recorded, which may possibly be attributed to an increase in the dispersed phase [34]. On the other hand, increasing the surfactant concentration while using same oil concentration resulted in lowering the formulation particle size. These findings are in accordance with Ren et al., who stated an inverse relationship between the nanoemulsion particle size and the surfactant concentration [35]. The following regression equations can illustrate the action of independent variables Y_1_ on the perceived response:

R_1_ = 189 + 41.7347 A − 10.7819 B − 6.75 AB + 4.3125 A^2^ − 3.6875 B^2^ (1)

**Figure 1:**
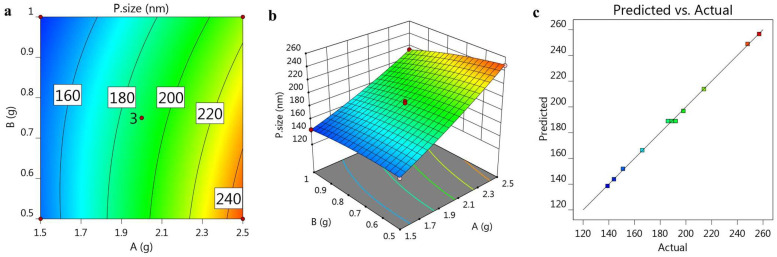
(**a**) 2D-contour plot, (**b**) 3D-response surface plot and (**c**) linear correlation plot between the predicted and actual values for investigating the influence of variables A and B on particle size (Y_1_).

As a general rule, the presence of a positive sign indicates a synergistic action, contrary to a negative one, which describes an antagonistic effect [36]. It is obvious from the previous equation that variable (A) showed a positive influence on particle size, although variable (B) showed a negative effect. Besides this, Figure 1a,b, represented by the 2D-contour and 3D-response surface plot, respectively, illustrate the effect of these variables, A and B, on response Y_1_. Moreover, as recorded in Table 3 and depicted in Figure 1c, there was an association between the adjusted R^2^ value (0.9972) and the predicted one (0.9958) for the particle size factor, which supports the linearity of the data, since the variation between the two values was less than 0.2. It was obvious that the system could recommend the model due to the R^2^ value (0.9986), in addition to the value of the adequate precision (77.2758), which showed an adequate signal, which concludes that the model could navigate the design space.

#### 2.3.2. Effect of Independent Variables on In Vitro Release Investigation

**Figure 2:**
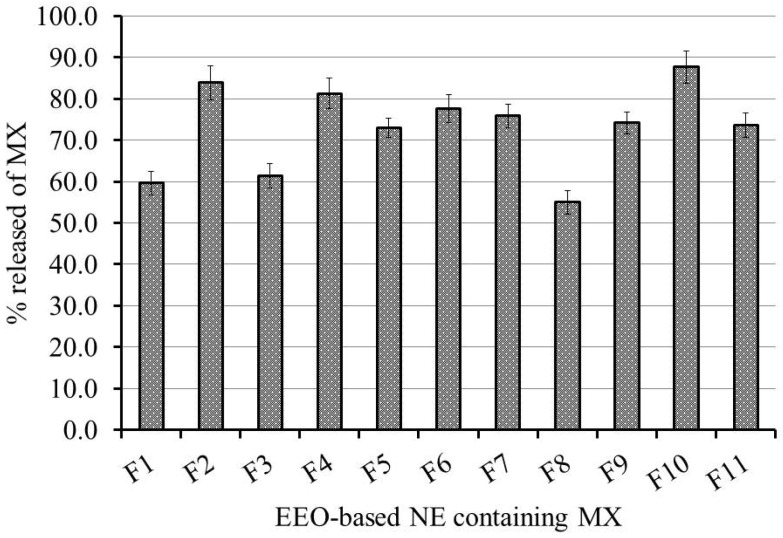
In vitro release of MX from various EEO-based NE formulations in a phosphate buffer with pH 7.4 at 37 °C. Results are presented as the mean ± SD of three experiments.

Studying the in vitro release of MX from the developed EEO-based NEs was effectively employed, and the outline of the release is portrayed in Figure 2. As shown in the figure, the percentage of MX released from the preparations following 6 h ranged between 55.0 ± 2.8% and 87.6 ± 3.9%. It is apparent that upon increasing the oil concentration in the preparation, it showed a decrease in the percentage of MX released from the NE. This could be attributed to the fact that a higher oil concentration would provide the NE with a greater particle size, which would offer a smaller surface area and, consequently, a lower percentage of drug released [37]. Conversely, it was distinguished that while using same concentration of oil, increasing the surfactant concentration would result in ameliorating the release of the drug from the NE, owing to enhancing the solubility of the drug in the aqueous media [38].

**Figure 3:**
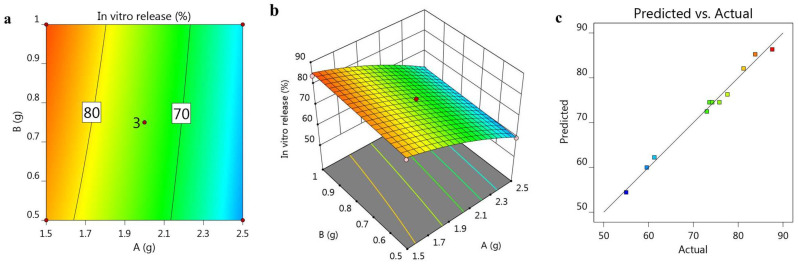
(**a**) 2D-contour plot, (**b**) 3D-response surface plot and (**c**) linear correlation plot between the predicted values and actual ones, representing the effect of variables A and B on the in vitro release study (Y_2_).

The previous in vitro release results are represented in Figure 3a, showing a 2D-contour, and Figure 3b, displaying a 3D-response surface plot. The influence of the independent variables on the inspected in vitro release was illuminated, since the oil concentration exerted an antagonistic action on the response, while the surfactant concentration exerted a synergistic effect. Moreover, Figure 3c displays the linearity of the value via a linear correlation plot, where the adjusted R^2^ value for the in vitro release factor was 0.9806, and the predicted one was recorded as 0.9428. As is apparent in Table 4, the values of the adjusted and predicted R^2^ showed that there was reasonable agreement between them, whereby the difference was less than 0.2. Additionally, the R^2^ value was 0.9903, and the adequate precision was 30.0023, which indicate that the model could navigate the design space. The following mathematical equation can express the association between the independent variables and their substantial influence on the responses:

R^2^ = 74.5333 − 11.2754 A + 1.35067 B − 0.225 AB − 2.07292 A^2^ − 0.0729167 B^2^ (2)

### 2.4. Optimizing the Independent Variables Using CCD

**Figure 4:**
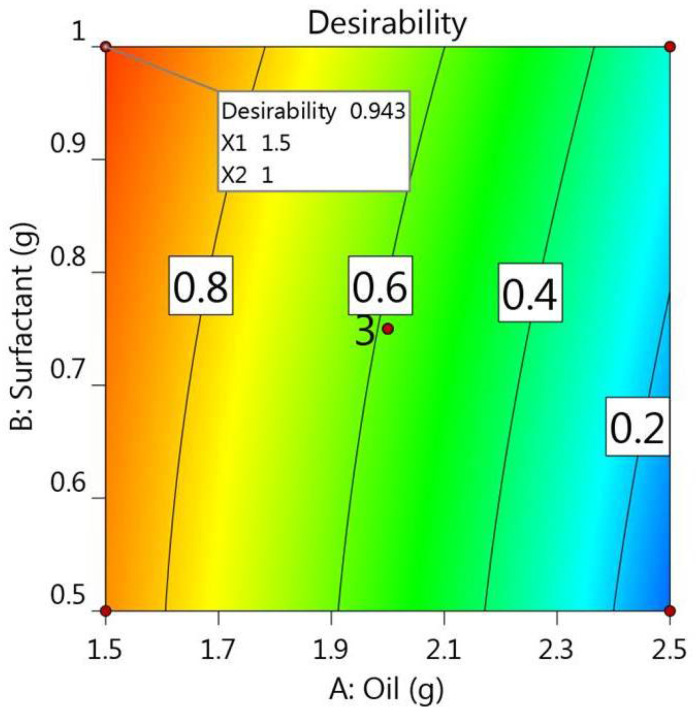
Desirability figure displaying the effect of the oil concentration and surfactant concentration on the overall responses.

**Figure 5:**
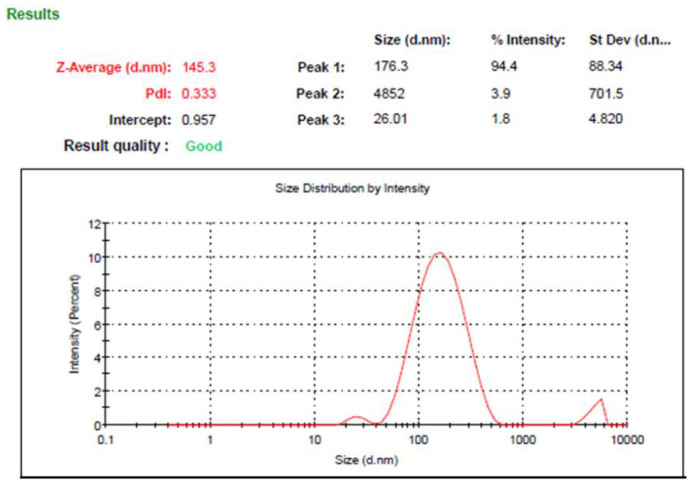
Particle size of the optimized EEO-based NE formulation incorporating MX.

The process of optimization is a very critical step in determining the best features and proper constraint level, depending on the higher value of desirability that results in a formulation with appropriate traits [39]. Numerical optimization was employed depending on the desirability function and the previous model graphs obtained from the design software. The selection of the optimized MX-NE formulation was accomplished after guiding responses to specific required goals. The target was to minimize the particle size while maximizing the percentage of the drug’s in vitro release. Based on that exploration, the independent variables were anticipated to be 1.5 g of oil and 1.0 g of surfactant. Moreover, and as apparent from Table 5, the software proposed certain data that were considered as the predicted values for the optimized formulation, and adding to that was the greater desirability value (0.943) that was attained, as shown in Figure 4. Relying on the suggested data of the independent variables, the optimized NE formulation was developed and matched with the observed calculated values. Significantly, it was revealed that both the predicted and calculated observed values were in agreement with each other, as displayed in the table. Figure 5 provides the distribution curve of the optimized MX-NE formulation, displaying its particle size (145.3 ± 3.7).

A new NE formulation was generated, depending on the predicted values of the CCD examinations, followed by further investigations. Figure 5 illustrates the particle size of the optimized MX-NE formulation.

In light of the results previously obtained, the optimized NE was integrated with the selected gelling agent through gentle stirring in order to develop a novel MX-NEG, which was subjected to definite additional studies.

### 2.5. Evaluating the MX-NEG

As obvious in Table 6, the MX-NEG prepared with HPMC was assessed with respect to several aspects and compared to the MX-G formulation. Following visual examination, the formulations seemed to be smooth and homogenous and showed an acceptable physical appearance. Relating to the pH value, it was measured and considered acceptable to prevent skin irritation. The drug-content evaluations seemed to be above 99.3%, indicating even distribution of the drug within the preparations. Concerning the viscosity, it was suitable to be easily applied over the skin. This was in addition to the spreadability assessment, which was revealed to be satisfactory. Broadly, certain evaluated parameters provided a significant difference between the MX-G and MX-NEG; however, all of the assessed properties of both formulations seemed to be reasonable and adequate for skin application.

### 2.6. In Vitro Release Study of Different Developed Formulations

**Figure 6:**
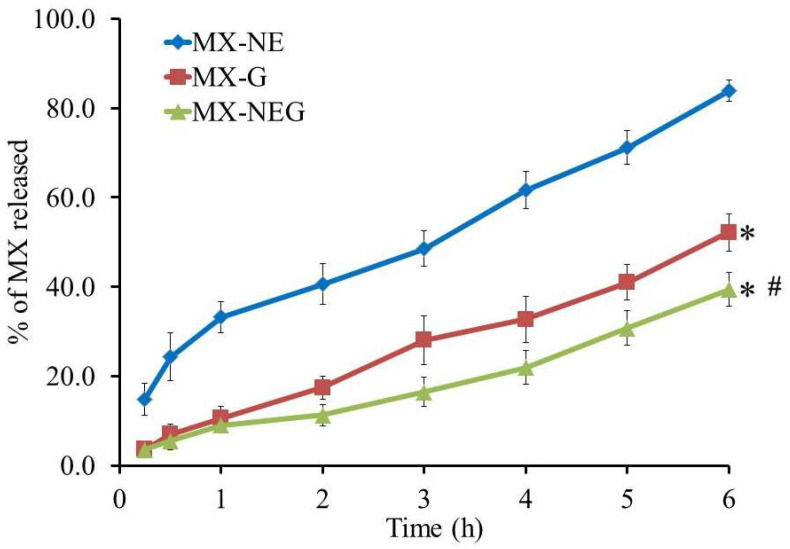
In vitro release of MX from the MX-NE, MX-G and MX-NEG formulation in a phosphate buffer with pH 7.4 at 37 °C. The results are expressed in respect to the mean ± SD of the three experiments. * *p* < 0.05 compared to the MX-NE formulation; # *p* < 0.05 compared to the MX-G formulation.

MX released from the developed gel and NEG formulations in a PBS with pH 7.4 over 6 h was assessed and compared to the optimized NE formulation, and the outline of the results is depicted in Figure 6. It was detected that the percentage of the drug released from the optimized EEO-based NE formulation was 83.9 ± 2.41%, which exhibited a significant difference with that released from the MX-G (52.1 ± 4.2%) and MX-NEG (39.4 ± 3.7%) (*p* < 0.05). It was previously reported that incorporating a gelling agent for attaining a gel or NEG exhibits great impacts on the drug’s release [40]. Actually, the viscosity of the developed formulation played a critical role in the percentage of the drug’s release, since a more viscous formulation showed a lower percentage of the drug’s release [41]. In the current investigation, the developed NEG formulation exhibited a higher viscosity than the MX-G and optimized NE formulation, owing to incorporating HPMC, which acted like a gelling agent. Remarkably, there was a significant difference (*p* < 0.05) observed between the percentage of MX released from MX-G and that released from MX-NEG (*p* < 0.05). That finding could be accredited to the aqueous content of the gel preparation, which accelerates the movement of the drug into the media of release [12]. Although NE showed better in vitro release results, NEG was considered to be more preferable as it achieved good adhesion, proper spreadability that led to better skin penetration and was practically easier to apply topically [42].

### 2.7. Stability Study of the Developed MX-NEG Formulation

**Figure 7:**
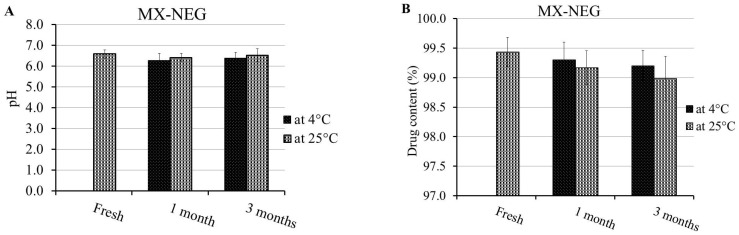

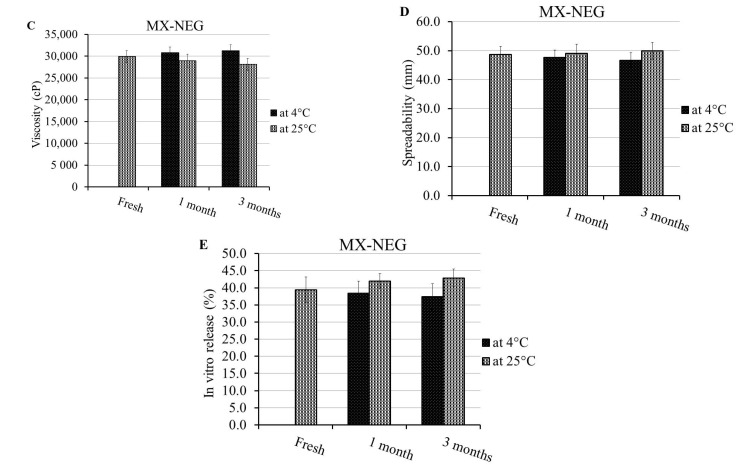
Stability profile of the MX-NEG formulation following 1 and 3 months at 4 °C and 25 °C in terms of (**A**) pH, (**B**) % of drug content, (**C**) viscosity, (**D**) spreadability and (**E**) % of in vitro drug release compared to a freshly prepared formulation.

Investigating the stability of the MX-NEG formulation was accomplished, and the data are exhibited in Figure 7A–E. The results were assessed over a period of 1 and 3 months and compared to a fresh NEG preparation. Non-significant variations were perceived in terms of the formulation’s character (pH, % of drug content, viscosity, spreadability) and the percentage of MX’s in vitro release from the stored formulation, which certified an excellent stability profile of the formulation and ensured its proficiency as a nanocarrier (*p* < 0.5).

### 2.8. Ex Vivo Permeation Study

**Figure 8:**
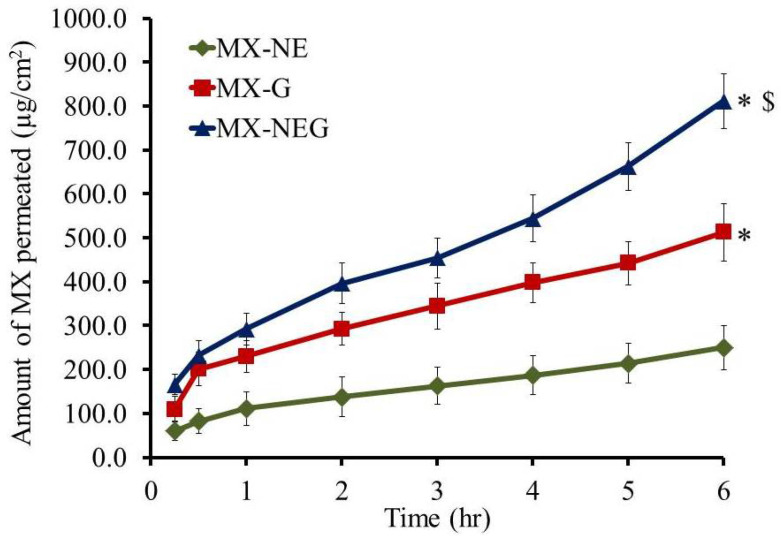
Permeation study of MX from different formulations across animal skin. Results are expressed as the mean ± SD (n = 3). * *p* < 0.05 compared to MX-NE; ^$^ *p* < 0.05 compared to the MX-G formulation.

Figure 8 exhibits the skin permeation outline of MX across rat skins from different examined formulations. Additionally, Table 7 demonstrates specific parameters associated with the ex vivo permeation study. Following 6 h, it was detected that a significantly larger amount of MX permeated from the NEG formulation, showing an SSTF value 141.28 ± 9.17 µg/cm^2^, compared to that that permeated from MX-G (84.28 ± 10.83 µg/cm^2^) (*p* < 0.05). It was confirmed that the permeation of MX-NEG was enhanced by 1.68 ± 0.11 fold compared to MX-G. Essentially, the greater permeation of the drug from the NEG formulation could be attributed to the small particle size of the main NE preparation. The incorporation of the drug into nanosized globules could facilitate the drug’s permeation through the skin layer [43]. Moreover, the integration of tween 80 and PEG 400 played a vital role in enhancing the MX flux from the NEG. Additionally, the external aqueous phase could hydrate the stratum corneum, which can facilitate the passage of the drug [44]. Besides this, the nano-size of NEG resulted in a larger surface area and, consequently, aa higher permeation of MX from the NEG formulation to the area of treatment [45]. Furthermore, the presence of transcutol^®^ P is regarded as an important point in skin permeability, since it behaves like an efficient penetration enhancer and provides better penetration to the stratum corneum. Osborne et al. reported that transcutol^®^ P resulted in increasing the drug concentration in the skin, which indicates that it acts like a skin penetration enhancer [46].

### 2.9. In Vivo Study

#### 2.9.1. In Vivo Skin Irritation Test

The animals treated with MX-NEG were checked on their back skin for any sensitivity reactions that might happen through conducting a skin irritation test. It was observed that no irritation, erythema or edema were identified on the examined area during the whole period of the study, which indicates the formulation’s safety.

#### 2.9.2. In Vivo Anti-Inflammatory Study: Carrageenan-Induced Rat Hind-Paw Edema Method

**Figure 9:**
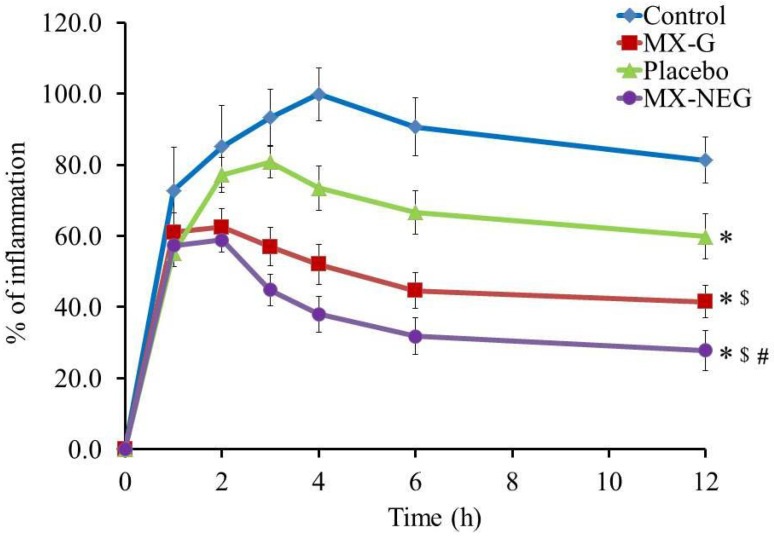
Representing the anti-inflammatory effects of various formulations on a rat hind-paw edema. Results are expressed as the mean with the bar showing the SD (n = 6). * *p* < 0.05 versus the control treated group; ^$^ *p* < 0.05 versus the placebo treated group; ^#^ *p* < 0.05 versus the MX-G-treated group.

The outcome of the anti-inflammatory investigation on the rat hind-paw induced with edema was obtained, as displayed in Figure 9. It was noted in all treated groups that the thickness of the edema was directly proportional to the percentage of inflammation. Following 4 h, the control treated group provided the greatest percentage of inflammation (99.8 ± 7.5%), while the placebo treated group displayed a significantly lower percentage of inflammation (80.8 ± 4.4%) after 3 h (*p* < 0.05) compared to the control group. Additionally, following 2 h of the investigation, the MX-G-treated group reached the highest percentage of inflammation (62.5 ± 5.2%), which showed no significant difference if compared to group treated with the nanoemulgel formulation (58.8 ± 3.5%) (*p* < 0.05). Moreover, following 12 h, it was notable that the percentage of inflammation was 81.3 ± 6.4, 59.8 ± 6.4, 41.5 ± 4.6 and 27.8 ± 5.7% for the control, placebo, MX-G- and MX-NEG-treated groups, respectively. It is worth mentioning the decrease in the percentage of inflammation caused in the placebo group, which confirms EEO’s role in combating inflammation. Furthermore, there was a significant difference (*p* < 0.5) between the MX-G- and MX-NEG-treated groups, which suggests that NEG possesses a considerable anti-inflammatory effect, owing to its greater permeability through skin [12]. In nutshell, the study evidences the anti-inflammatory effect of EEO and its synergistic action with MX.

## 3. Conclusions

In the current investigation, eucalyptus essential oil-based nanoemulsion formulations incorporating meloxicam as an anti-inflammatory agent were efficiently developed, characterized and optimized via a central composite design software system. The best nanoemulsion formulation was selected and integrated with a gelling agent to attain a nanoemulgel formulation. The developed nanoemulgel exhibited an appropriate pH, drug content, viscosity and spreadability, which was considered satisfactory for topical application. An in vitro release and skin permeation study was commendably performed over 6 h, confirming reasonable results if compared to the gel formulation. Finally, the anti-inflammatory effect of the nanoemulgel was synergistically enhanced when combining meloxicam with eucalyptus essential oil. 

## 4. Materials and Methods

### 4.1. Materials

Meloxicam was obtained from Sigma-Aldrich Co. (St. Louis, MO, USA). Eucalyptus essential oil was obtained from NOW^®^ Essential Oils (NOW Foods, Bloomingdale, IL, USA). Diethylene Glycol Monoethyl Ether (Transcutol^®^ P) was procured from Gattefosse SAS (Saint-priest Cedex, Lyon, France). Polysorbate 80 (Tween 80), Hydroxpropyl methylcellulose (HPMC) (K15M) and poly ethylene glycol 400 (PEG 400) were purchased from Sigma-Aldrich Co. (St. Louis, MO, USA). All other chemicals were of the finest grade available.

### 4.2. Solubility Examination of MX in the Formulation Constituents 

The solubility of MX was assessed in EEO, tween 80, PEG 400 and transcutol^®^ P. An excess amount of MX was shaken with 1 mL of each component separately for 48 h at 25 °C, using a shaker water bath (Gesellschaft fur Labortechnik mbH, Burgwedel, Germany). Next, samples were centrifuged for 15 min at 2000 rpm using a centrifuge (Andreas Hettich GmbH, Co.KG, Tuttlingen, Germany); then, they were filtered and diluted with methanol. The samples were analyzed spectrophotometrically (U.V. Spectrophotometer, JENWAY 6305, Bibby Scientific Ltd., Stone, Staffs, UK) at λ_max_ of 360 nm [30].

### 4.3. Experimental Design

A two-factor, two-level (2^2^) factorial design was implemented in order to optimize the developed NE formulations using Response surface methodology (RSM), which was mainly CCD. Two factors representing the independent variables were selected (oil concentration, A, and surfactant concentration, B), which were examined at two levels, low (−1) and high (+1), as shown in Table 8. The influence of these factors on the response of the prepared NE was investigated, such as particle size (Y_1_) and in vitro drug release (Y_2_). The Design-Expert version 12.0 software (Stat-Ease, Minneapolis, MN, USA) was employed for carrying out the current optimization via generating statistical analyses of the data using an Analysis of variance (ANOVA) test. Subsequently, modeling graphs were constructed in addition to mathematical equations that provide an illustration for the response as follows:

Y = b_0_ + b_1_A + b_2_B + b_12_AB + b_11_A^2^ + b_22_B^2^ (3)

where Y denotes the selected response, and b_0_ denotes the intercept; b_1_, b_2_, b_12_, b_11_ and b_22_ are the regression coefficients. A and B characterize the studied factors; AB symbolize the interactions between the main factors, while A^2^ and B^2^ indicate the polynomial terms.

### 4.4. Development of an EEO-Based NE Loaded with MX

Various NE preparations incorporating MX were developed using the specified amounts of constituents. A total of 1% (*w*/*w*) of MX was added into the specified amount of EEO and transcutol^®^ P (0.5 g), which is an excellent solvent, and was well-mixed to form the oily phase. Different amounts of tween 80 acting as a surfactant and PEG 400 (0.5 g) as a co-surfactant were added to distilled water, followed by vortexing to form the aqueous phase. Both phases were mixed together, and the volume was adjusted to reach 10 g with distilled water. Mixing of the two phases continued for 15 min using a high shear homogenizer (T 25 digital Ultra-Turrax, IKA, Staufen, Germany) at 20,000 rpm. Promptly, the NE was formed next to homogenization; afterward, it was subjected to 1 min of sonication using a probe sonicator (XL-2000, Qsonica, Newtown, CT, USA) [47]. The matrix of 11 experimental formulations was constructed using CCD along with the values of their observed responses, as clarified in Table 1.

### 4.5. NE Characterization

#### Particle size and Polydispersity Index (PDI) Determination

A Zetasizer apparatus (Malvern Instruments Ltd., Worcestershire, UK) was operated in order to analyze the particle size and PDI of all of the generated MX-NE formulations. These characters were measured using dynamic light scattering, which was adjusted at 25 °C, using a scattering angle of 90° [48].

### 4.6. In Vitro Release

An Agilent Fiber optics dissolution system (Agilent Technologies, San Francisco, CA, USA) was run to determine the percentage of MX released from all of the generated NE formulations. Glass tubes were used in the system as substitutes to the baskets. The tubes were sealed from one side with a cellophane membrane (MWCO 2000–15,000), to which 2 mL of the preparation was added. A vehicle of 500 mL of phosphate buffer saline (PBS) of pH 7.4 was added into the system and kept at 37 ± 0.5 °C and then rotated at 50 rpm. At definite intervals of time (0.25, 0.5, 1, 2 and 6 h), the samples were examined at λ_max_ of 360 nm. The same procedure was performed to measure the percentage of MX released from the developed NEG formulation [49].

### 4.7. Development of MX-NEG

MX-NEG was fabricated by incorporating a gelling agent into the optimized EEO-based NE loaded with MX. Approximately 4% *w*/*w* of HPMC, which behaves as a gelling agent, was steadily scattered into 10 mL of distilled water to provide an HPMC hydrogel and was then mixed with an EEO-based NE containing the drug. The mixture was well-mixed for 5 min using Heidolph RZR 1 (Heidolph Instruments, Schwabach, Germany) in order to obtained consistent NEG (20 g) [50]. For a distinctive evaluation of the MX-NEG efficacy, a placebo NEG (without MX) was developed using same procedure of preparing MX-NEG and the same amount of constituents (EEO (1.5 g), Tween 80 (1.0 g), PEG 400 (0.5g) and transcutol^®^ P (0.5 g)). Moreover, the MX gel (MX-G) was fabricated by scattering MX over 2% HPMC gel.

### 4.8. Evaluating the Developed MX-NEG

#### 4.8.1. Physical Examination

The prepared MX-NEG formulation was visually examined for its appearance, color and homogeneity.

#### 4.8.2. pH Measurement

A standardized pH meter (MW802, Milwaukee Instruments, Szeged, Hungary) was utilized to determine the formulation’s pH and confirm its safety to be applied topically on the skin [51].

#### 4.8.3. Drug Content

A precise amount of the NEG preparation (1 g) was diluted in 100 mL of phosphate buffer saline (PBS) and then filtered using 0.45 micro-syringe filters. The drug content was assayed spectrophotometrically at an λmax of 360 nm [52]. For the blank sample (sample without the drug), the identical technique was carried out, and then, the drug content was measured using the following equation: Drug content = (Actual/Theoretical) × 100.

#### 4.8.4. Viscosity

In order to measure the viscosity of the developed MX-NEG formulation, a Brookfield viscometer (DV-II+ Pro, Middleboro, USA) was operated at 25 °C, using spindle R5, and allowed to rotate at 0.5 rpm [49].

#### 4.8.5. Spreadability Test

The spreadability of the formulation is an indicator of its capability to spread readily when applied on the skin by determining the spreading diameter. Concisely, a certain amount of the NEG formulation (1 g) was held in-between two glass slides (25 cm × 25 cm), and a certain load was fixed over the system for 1 min. The value of the spreadability was obtained by measuring the spreading diameter of the formulation over the affected area [53].

### 4.9. In Vitro Release Study from Different Developed Formulations

The percentage of the drug released from the different prepared formulations was estimated via an Agilent Fiber optics dissolution system (Agilent Technologies, San Francisco, CA, USA). Definite amounts of the optimized EEO-based NE, MX-G and MX-NEG formulations were examined, whereby the same technique mentioned previously in Section 2.5 was followed. 

### 4.10. Stability Study of MX-NEG

The developed MX-NEG was verified for its stability with regards to the evaluated characterization parameters, including physical examination, pH, drug content, viscosity, and spreadability, in addition to the in vitro release study. These factors were measured after being stored for 1 and 3 months at two different conditions: 4 ± 1 °C and at 25 ± 1 °C. This exploration was performed in accordance with the guidelines of the International Conference on Harmonization (ICH) [54].

### 4.11. Ex Vivo Study 

#### 4.11.1. Preparation of Rat Skin

In the present study, the skin of Male Wister rats was utilized. First, an electric clipper was used to carefully remove the animal’s dorsal hair. Then, the rats were sacrificed, and the skin was cut out. The skin was hydrated overnight in a phosphate buffer (pH 7.4) at 4 °C after removing the adipose tissue [50].

#### 4.11.2. Permeation Study

**Figure 10:**
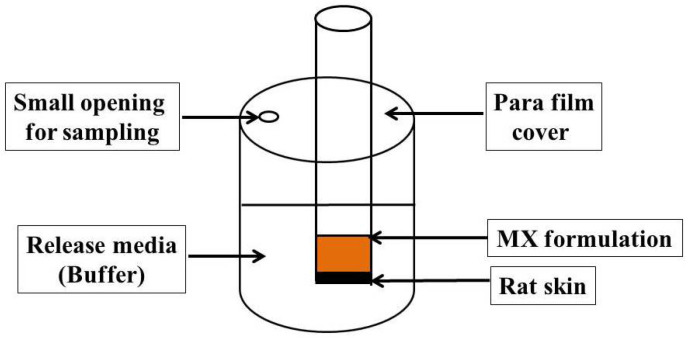
Adapted Franz diffusion cell for the permeability study of MX formulations across rat skin.

Adapted Franz diffusion cells established in our lab were employed to detect the permeation of MX from the gel and NEG through the skin of male Wister rats, as illustrated in Figure 10 [55,56,57]. The diffusion system was kept at 37 ± 0.5 °C and contained 100 mL of phosphate buffer (pH 7.4) with 0.02% sodium azide. Glass tubes were suspended in the media in the apparatus. The rat skin was attached to the diffusion cell, wherein the upside of the skin faced the formulation, while its dermis was in front of the receptor media. The examined formulation was enclosed with the rat skin membrane and fixed to the glass tubes. In order to evade evaporation of water, the cells were covered with Parafilm (Bemis, Oshkosh, WI, USA), and the system was stirred at 100 rpm. [58]. The steady state transdermal flux (SSTF) and enhancement ratio (ER) are two parameters relating to the ex vivo permeation study that were evaluated, since SSTF represents the amount of permeated drug/(area × time); whereas ER denotes SSTF from the test/SSTF from the control.

### 4.12. Animal

Aiming to carry out animal experiments, 220–250 g Male Wister rats were supplied from an animal breeding center, from the College of Science, King Faisal University. Animals were maintained under a controlled housing condition, in which the light and dark cycle was adjusted to be 12:12 h, and at an ambient temperature (25 ± 2 °C). Regarding the ethical statement, all experiments were conducted in accordance with the guidelines and were ethically approved by the Research Ethics Committee (REC) of King Faisal University, approval number KFU-REC-2021-DEC-EA000308.

### 4.13. In Vivo Study

#### 4.13.1. In Vivo Skin Irritation Test

It is very important to guarantee the safety of the formulation, especially if intended for topical application. This study was conducted using male Wister rats by preparing them one day earlier. Then, the dorsal hair of the animals was shaved using an electric clipper, and the examined formulation (MX-NEG) was evenly spread over the shaved area. The animals were observed for 7 days after being treated topically with the formulation in order to detect any responses relating to irritation erythema (redness) or edema. Any perceived response was demonstrated based on a scale that varied from 0 to 3, which signified that the sensitivity reactions could be no, a minor, a moderate or a severe erythema reaction, with or without edema [6].

#### 4.13.2. In Vivo Anti-Inflammatory Study: Carrageenan-Induced Rat Hind Paw Edema Method

The EEO and MX anti-inflammatory activity was evaluated by performing the carrageenan-induced rat hind-paw edema model using male Wister rats. This method was conducted based on former reports adopted from Shehata et al. [59], wherein an edema was induced in rat hind-paws half an hour prior to drug administration by means of injecting 0.5% *w*/*v* carrageenan in saline subcutaneously into the left hind paw [60]. Rats were divided randomly into four groups, where n = 6, as follows: 

Group I was the untreated group (control group).

Group II was treated with MX-G (5.6 mg/kg).

Group III was the placebo group, which was treated with NEG free from the drug. 

Group IV was treated with MX-NEG (5.6 mg/kg) [61]. 

At varied time intervals (0, 1, 2, 3, 4, 6 and 12 h), the inflammatory reaction was evaluated. This was accomplished using a digital caliber to detect the variations in the volume of the rat paw next to the transdermal application. The following equation was utilized to validate the % of inflammation:

% of inflammation = ((Vt − V0)/V0) × 100 (4)

where Vt denotes the volume of the carrageenan-treated hind paw, whereas V0 characterizes the hind paw at time zero.

### 4.14. Statistics

All experiments were confirmed by performing at least three independent trials as the mean ± SD. A student’s *t*-test was employed to detect the statistical differences between the groups. A one-way analysis of variance (ANOVA), followed by the least significant difference (LSD) as a post-hoc test, was applied to compare data from the treated and control groups. These evaluations were established using SPSS statistics software, version 9 (IBM Corporation, Armonk, NY, USA). A statistically significant difference between groups was approved if *p* < 0.05.

## Figures and Tables

**Table 1:** Experimental design and value of each independent variable and observed response for different nanoemulsion preparations.

Formula	Independent Variables		Response Values	
	A (g)	B (g)	Y_1_ (nm)	Y_2_ (%)
F1	2.5	0.50	248 ± 3.21	59.6 ± 2.9
F2	1.5	1.0	144 ± 3.02	83.8 ± 4.2
F3	2.5	1.0	214 ± 4.04	61.3 ± 3.0
F4	1.5	0.50	151 ± 2.89	81.2 ± 3.7
F5	2.0	0.39	198 ± 4.36	73.0 ± 2.4
F6	2	1.1	166 ± 3.69	77.6 ± 3.5
F7	2	0.75	186 ± 3.06	75.8 ± 2.9
F8	2.7	0.75	257 ± 3.61	55.0 ± 2.8
F9	2.0	0.75	189 ± 2.00	74.2 ± 2.7
F10	1.29	0.75	139 ± 2.31	87.6 ± 3.9
F11	2.0	0.75	192 ± 2.65	73.6 ± 3.1

A: Oil concentration; B: Surfactant concentration; Y_1_: particle size and Y_2_: In vitro release.

**Table 2:** Statistical analysis of responses.

Source	Y_1_		Y_2_	
	F-Value	*p*-Value	F-Value	*p*-Value
Model	715.45	<0.0001 *	102.14	<0.0001 *
A	3257.21	<0.0001 *	490.98	<0.0001 *
B	217.39	<0.0001 *	7.05	0.0452 *
AB	42.60	0.0013 *	0.0978	0.7672
A²	24.55	0.0043 *	11.71	0.0188 *
B²	17.95	0.0082 *	0.0145	0.9089
Lack of Fit	0.1255	0.9369	2.00	0.3501

A, oil concentration (g); B, surfactant concentration (g); Y_1_, particle size (nm); Y_2_, In vitro release (%); *, significant.

**Table 3:** Regression analysis and model summary statistics for the final suggested model.

Dependent Variable	Source	R²	Adjusted R²	Predicted R²	SD	Adequate Precision	Remark
Y_1_	Linear	0.9700	0.9624	0.9322	7.59	-	-
	2FI	0.9818	0.9741	0.9592	6.30	-	-
	Quadratic	0.9986	0.9972	0.9958	2.07	77.2758	Suggested
	Cubic	0.9988	0.9958	0.9927	2.52	-	-

**Table 4:** Regression analysis and model summary statistics for the final suggested model.

Dependent Variable	Source	R²	Adjusted R²	Predicted R²	SD	Adequate Precision	Remark
Y_2_	Linear	0.9657	0.9572	0.9312	2.14	-	-
	2FI	0.9659	0.9513	0.8989	2.28	-	-
	Quadratic	0.9903	0.9806	0.9428	1.44	30.0023	Suggested
	Cubic	0.9913	0.9711	0.5955	1.76	-	-

**Table 5:** Predicted and observed values for the optimized MX-nanoemulsion formulation.

Dependent Variable	Symbol	Constraint
Oil concentration	A	In range
Surfactant concentration	B	In range
Response	Predicted values	Observed values
Y_1_ (nm)	143.85 ± 2.06	145.3 ± 3.7
Y_2_ (%)	85.23 ± 1.43	83.9 ± 2.41

**Table 6:** Properties of the MX-NEG formulation.

Character	MX-G	MX-NEG
Visual examination	Smooth and homogenous	Smooth and homogenous
pH	6.4 ± 0.2	6.58 ± 0.21
Drug content (%)	99.3 ± 0.46	99.4 ± 0.25
Viscosity (cP)	11,580 ± 775.8	29,920 ± 1373.9 *
Spreadability (mm)	57.4 ± 2.3	48.6 ± 2.9 *

Data are expressed as the mean ± (SD) using a student *t*-test. * *p* < 0.05 compared to MX-G.

**Table 7:** Skin permeation parameters of the different developed formulations.

Formula	SSTF µg/cm^2^·h	ER
MX-G	84.28 ± 10.83	1
MX-NEG	141.28 ± 9.17 *	1.68 ± 0.11 *

Values are expressed as the mean ± SD. * *p* < 0.05 compared to MX-G.

**Table 8:** Independent variables and their level of variation.

Independent Variable	Symbol	Level of Variation	
		(–1)	(+1)
Oil concentration (g)	A	1.5	2.5
Surfactant concentration (g)	B	0.5	1.0

## Data Availability

Not applicable.
